# Prospective association of family members’ sugar-sweetened beverages intake with children’s sugar-sweetened beverages consumption in China

**DOI:** 10.1007/s00394-022-02971-3

**Published:** 2022-08-05

**Authors:** Xue-Ting Liu, Jing-Yuan Xiong, Yu-Jie Xu, Li Zhao, Lars Libuda, Guo Cheng

**Affiliations:** 1 Department of Nutrition and Food Safety, West China School of Public Health and West China Fourth Hospital, Sichuan University, Chengdu, China; 2 Healthy Food Evaluation Research Center, West China School of Public Health and West China Fourth Hospital, Sichuan University, Chengdu, Sichuan China; 3 Laboratory of Molecular Translational Medicine, Center for Translational Medicine, Key Laboratory of Birth Defects and Related Diseases of Women and Children (Sichuan University), Ministry of Education, West China Second University Hospital, Sichuan University, Chengdu, Sichuan China; 4 Department of Pediatrics, West China Second University Hospital, Sichuan University, Chengdu, Sichuan 610041 People’s Republic of China; 5 Department of Health Policy and Management, West China School of Public Health and West China Fourth Hospital, Sichuan University, Chengdu, Sichuan China; 6 Faculty of Natural Sciences, Institute of Nutrition, Consumption and Health, Nutrition Sciences, Paderborn University, 33098 Paderborn, Germany

**Keywords:** Sugar-sweetened beverage, Children, Parent, Sibling, Sex difference

## Abstract

**Purpose:**

We aimed to investigate whether parental and siblings’ sugar-sweetened beverage (SSB) intake had prospective impact on children’s SSB consumption, and the potential sex difference in these associations.

**Methods:**

This study included a total of 904 children and their parents enrolled from 2004 to 2011 China Health and Nutrition Survey (CHNS) cohort study. SSB consumption information was estimated using a short dietary questionnaire and total energy intake was assessed with three-day 24-h dietary assessments at recruitment and follow-up surveys. Multivariate logistic or linear regression analyses were used to assess the association for SSB consumption between parents, siblings and children after adjusting for age, body mass index (BMI) *z*-score, household income and parental educational level.

**Results:**

In this study, a majority (87.6%) of children consumed SSB. Among them, the median consumption of SSB was 70.3 ml/day per capita and 205.4 ml/day per consumer. Parental SSB consumption was relevant to children’s SSB consumption, and this association was more pronounced in boys than in girls. Meanwhile, fathers seemed to have a stronger impact on whether children consume SSB than mothers which was reflected by lower *P* and higher OR. Additionally, children’s SSB intake was prospectively associated with their older siblings’ SSB consumption (*P*_for trend_ < 0.03).

**Conclusions:**

Parental and older siblings’ SSB consumption was relevant to children’s SSB intake. Particularly, boys were more susceptible to parental impact than girls, and fathers seemed to have a greater influence on children than mothers.

**Supplementary Information:**

The online version contains supplementary material available at 10.1007/s00394-022-02971-3.

## Introduction

Over the last 20 years, epidemiological studies have indicated a 24.4% increment of sugar-sweetened beverage (SSB) consumption among Chinese children [1]. Higher SSB consumption is an established risk factor for childhood obesity and dental caries [2] and is suggested to be associated with cancer [3], cardiovascular disease and metabolic syndrome later in life [4]. Thus, to protect children from health problems and health hazards, more attention is required to address the causes and factors influencing children’s SSB consumption.

Family factors have been proved to be one of the main influencing factors affecting children’s SSB consumption, and associations between family members’ SSB consumption and children’s diet with high SSB intake have been found in cross-sectional studies [1, 5–13]. While the correlation is strong, it is not possible to unequivocally determine the direction of causality. A bidirectional relationship may exist between adolescents and family members [14]. Thus, prospective studies with a clearly defined temporal relationship are more convincing than cross-sectional studies in elucidating potentially causal links between family members’ SSB consumption and children’s SSB intake. Moreover, sex differences in intergenerational relationships between parents and offspring were found in alcohol consumption [15] and physical activity [16], and sex difference also exists in SSB drinking behavior [17]. These lead us to speculate on the possible sex differences in the association for SSB intake between parents and children, which did not attract widespread attention yet.

Among family members, an important area of study is the extent to which the SSB consumption patterns of adults influence those of children in their households. Most studies that describe SSB consumption within families assess one child with one parent [5, 6, 8, 10–12] or guardian [7, 9]. It is argued that fathers and mothers provided different experiences for adolescents’ health status [18]. Only conducted in one parent may induce selection bias. In addition to parents, siblings could be another influencing factor for children to establish health attitudes toward physical activity[19] and dietary habits [20], such as eating frequency and food diversity. To date, there is only one cross-sectional study in Great Britain that indicated the role of siblings on children’s SSB consumption [13]. And this association, to our knowledge, has never been explored thus far in China.

Considering the secular trend of increasing SSB intake in Chinese children and its short- and long-term health concerns, this study aimed to investigate whether family members’ SSB consumption was prospectively associated with children’s SSB intake, including: (1) parental impact on children’s SSB consumption, and potential sex difference; (2) sibling’s impact on children’s SSB intake.

## Subjects and methods

### China health and nutrition survey

China health and nutrition survey (CHNS), an ongoing household-based open cohort, has been conducted 11 waves in 15 provinces and municipal cities in China between 1989 and 2015. In brief, CHNS uses a multistage random cluster sampling method to select a demographically representative Chinese sample. All participants provided written informed consent in the survey [21] and could join or withdraw from the study at any survey wave. The study protocol was approved by the ethical review committees of the Chinese Center for Disease Control and Prevention and the University of North Carolina at Chapel Hill. Detailed description of CHNS design can be found on its official website [22].

### Study sample

**Fig. 1:**
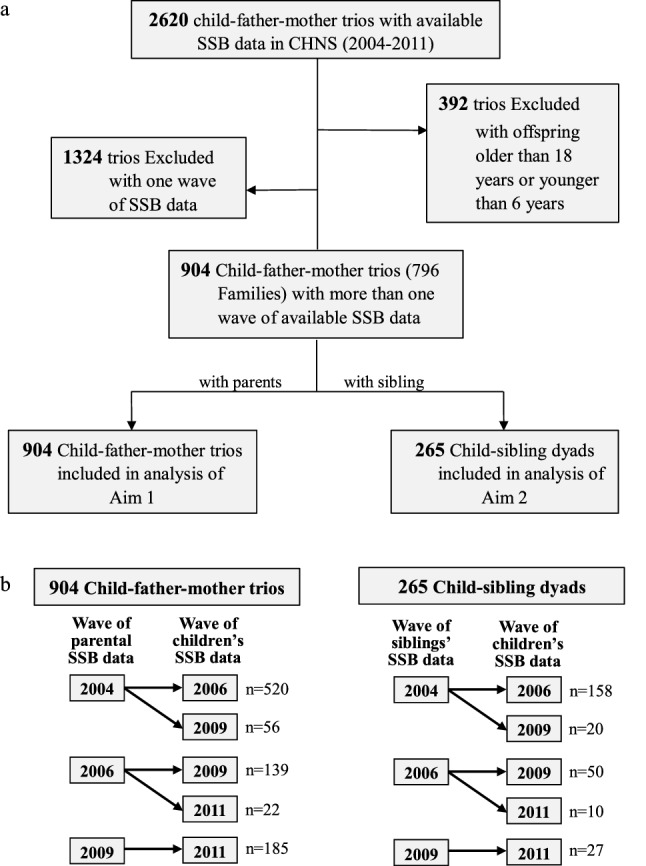
Data selection of study sample. a Flowchart for the selection of the analyzed study sample. **b** Chronological order of data collection from children to parents and siblings. SSB data of children were collected in 2006, 2009 and 2011, and SSB data of parents and siblings were traced back 2–5 years from children’s SSB data. And if parents or siblings had multiple waves of SSB consumption data, only data from the wave closest to the wave of children’s SSB data were retained

Data obtained from the China Health and Nutrition Survey are publicly available [23]. Since information on SSB consumption was collected in 2004, 2006, 2009 and 2011, the present analysis is based on these four waves of the survey. By the end of 2011, a total of 2620 offspring–father–mother trios provided data on SSB intake. Among them, offspring–father–mother trios (1) with offspring’s age was above 18 years old or less than 6 years (*n* = 392), or (2) with one wave of SSB data (n = 1324) were excluded. There were only small differences in the general characteristics of the included and excluded subjects (Table S1). In total, 904 child–father–mother trios were included in the analysis of the association for SSB consumption between parents and children, and 265 child–sibling dyads were included in the analysis of the association between siblings and children (Fig. 1a). A post-hoc power test (SAS proc power procedure) showed that the power was > 0.999 for the parental impact on children’s SSB consumption, and 0.776 for the sibling’s impact on children’s intake. The values were comparable to the criteria (0.8) introduced by Cohen [24]. To assure the exact chronological order of data collection, SSB data of children were collected in 2006, 2009 and 2011, and SSB data of parents and siblings were traced back 2–5 years for children’s SSB data (Fig. 1b). If parents or siblings had multiple waves of SSB consumption data, only data from the wave closest to the wave of children’s SSB data were retained.

### Dietary assessment

SSB consumption data were collected by trained personnel in personal interviews using a short dietary questionnaire from a structured individual questionnaire [25]. Children aged 6–10 years provided information with the help of parents or guardians, and others provided information on their own. SSB intake was obtained by these questions, ‘In the last year, did you drink SSB, and how much SSB did you drink each week?’. According to CHNS procedure, SSB was defined as Coca-Cola, soda, lemonade and juices with no more than 10% pure fruit juices [26].

Since complete dietary information is unavailable in the short dietary questionnaire, data on total energy intake was obtained from valid three consecutive 24-h recalls [27]. According to CHNS regulations, parents and children aged 12 years and older were asked to recall all foods and beverages they consumed. For children below 12 years, their parents or guardians provided information on food consumption at home, while children provided information on dietary intake away from home. The majority of participants (99.7%) completed three-day 24-h recalls (i.e., 42.4% had three weekdays, 29.0% had two weekdays and one weekend day, 18.7% had one weekday and two weekend days, and 9.6% had three weekend days) and only 3 children (0.3%) finished two-day 24-h recalls (i.e., 2 children had two weekdays, and 1 child had one weekday and one weekend day).

### Socio-demographic characteristics

Detailed information on socio-demographic characteristics was collected using a structured questionnaire at baseline, including sex, age (continuous variable), residency (urban and rural), education of father or mother (< 6 years, 6–12 years, > 12 years of schooling) and household monthly personal income (≤ 1800 CNY (Chinese Yuan), 1800–3200 CNY, ≥ 3200 CNY) [28].

Children aged 6–17 years were classified either as single child or child with sibling(s) after checking the matched household ID and parent ID in the CHNS dataset.

Anthropometric measurements for children were measured by the trained investigators following standard procedures [29]. Height was measured to the nearest 0.1 cm using a tape measure (Mechanical Measuring Tape No. 206; SECA) without shoes, and weight was measured to the nearest 0.1 kg using a weight scale (Floor Weight Scale No. 877; SECA, UK) with lightweight clothing [30]. In this analysis, body mass index (BMI) was calculated as weight divided by height squared (kg/m^2^). Age-specific BMI *z*-scores were calculated based on the Chinese children’s reference curves [31].

### Statistical analysis

Statistical Analyses System procedures (SAS, version 9.3, 2011, NC, USA) were used for all data analyses. Missing values (< 3%) of socio-demographic characteristics were substituted by mean and mode to substitute in the continuous and categorical variables, respectively [32]. A *P*-value < 0.05 was considered statistically significant, except for the interaction, where *P*-value < 0.1 was considered significant.

For categorical variables (sibship, income, residency and SSB consumption), frequencies were calculated. For continuous variables, BMI *z*-score was presented as means and standard deviation due to normal distributions; age and amounts of SSB consumption for consumers were presented as median and quartiles due to non-normal distributions. To obtain more information about the variation, the amount of SSB consumption for all participants was presented as mean (fifth percentile, ninety-fifth percentile) [33]. Differences in absolute amounts of SSB consumption, age, anthropometric data, sibship, income and residency between boys and girls were tested by Chi-square test for categorical variables, Student’s *t*-test for normally distributed continuous variables, and Kruskal–Wallis test for non-normally distributed continuous variables. Differences in the proportion of SSB consumers between the only-child and those with sibling(s), grouped by sex and parental SSB consumption, were tested by Fisher’s exact test.

To investigate the relevance of paternal, maternal or parental SSB consumption with offspring’s SSB intake (consume or not) across sex, multivariable logistic regression models were used. Parental SSB consumption was divided into three groups: (1) neither consumes (father and mother both do not drink SSB); (2) only one consumes (father or mother drink SSB); and (3) both consume (father and mother both drink SSB). In the basic models, paternal/maternal/parental SSB consumption were an independent predictor, respectively. In the adjusted model, variables potentially affecting this relevance were considered: age of offspring, BMI z-score, household monthly personal income, residency, sibship, age of parent, and parental educational level. In sensitive analysis, to test the robustness of our results, we repeated the logistic analysis within the participants without missing values on socio-demographic characteristics.

Among dyads with parents and children both consuming SSB, the relevance of parental amount of SSB intake on children’s amount of SSB consumption was tested using multivariable linear regression. Energy-adjusted (nutrient residual) model was used to adjust parents’ and children’s SSB consumptions [34]. The adjusted models were constructed in analogy to the multivariable logistic regression analyses. Multicollinearity was not found between independent variables and the socio-demographic characteristics (variance inflation factors < 3).

To investigate the prospective associations for SSB intake (consume or not) between siblings and children, multivariable logistic regression models were used. First, in the basic model, elder siblings’ SSB intake was regarded as an independent predictor. The sex of the younger child, the age difference between the two children in a household, and parental SSB consumption were considered as potentially affecting variables in the adjusted model. Second, younger siblings’ SSB intake was considered as an independent predictor in the basic model. This relevance was examined with the adjustment for the sex of elder children, the age difference between two children in a household, and parental SSB consumption in the adjusted model.

## Results

### Participant characteristics

The characteristics of 904 children are presented in Table 1. At the baseline, children had a median age of 12 (9, 14), and a mean BMI *z*-score of − 0.4 ± 1.2. There were slightly more boys (53.3%) than girls in this study. Most of the participants were only child and more than 85% of the participants were SSB consumers. Compared to children, a lower proportion of parents were SSB consumers and they also had a lower absolute amount of SSB intake. Among all participants, the mean intake of SSB was 70.3 ml/day for children, 23.2 ml/day for fathers and 19.7 ml/day for mothers. Among SSB consumers, the median intake of SSB was 205.4 ml/day for children, 35.7 ml/day for fathers and 21.4 ml/day for mothers.

**Table 1:** Baseline characteristics and SSB consumption of study sample (*n* = 904)^a^

Characteristics	Value
Socio-demographic issues	
Age at baseline (years)	12 (9, 14)
BMI z-score^b^	− 0.4 (1.2)
Boy [n (%)]	482 (53.3)
Single child^c^ [*n* (%)]	655 (72.5)
Paternal age (years)	39 (36, 42)
Maternal age (years)	38 (35, 41)
Parental education duration > 12 years [*n* (%)]	107 (11.8)
High monthly personal income^d^ [*n* (%)]	307 (34.0)
Live in urban area [*n* (%)]	305 (33.7)
Boy’s energy intake (kcal)	1714.0 (1397.7, 2186.2)
Girl’s energy intake (kcal)	1618.7 (1259.6, 1919.7)
Children’s SSB consumption	
Proportion of SSB consumers [*n* (%)]	792 (87.6)
SSB intake per capita^e^ (ml/day)	70.3 (0, 345.2)
SSB intake per consumers^f^ (ml/day)	205.4 (128.6, 300.0)
Paternal SSB consumption	
Proportion of SSB consumers [*n* (%)]	331 (36.6)
SSB intake per capita^e^ (ml/day)	23.2 (0, 107.1)
SSB intake per consumers^f^ (ml/day)	35.7 (17.9, 69.6)
Maternal SSB consumption	
Proportion of SSB consumers [*n* (%)]	437 (48.3)
SSB intake per capita^e^ (ml/day)	19.7 (0, 78.6)
SSB intake per consumers^f^ (ml/day)	21.4 (14.3, 37.5)

*SSB* sugar-sweetened beverage

^a^Values are means (SD) or medians (Q1, Q3) or frequencies

^b^BMI *z*-score, body mass index *z*-score calculated according to the Chinese reference curves[31]

^c^Single child, children from families with only one child

^d^Monthly personal income at least ≥ 3200 CNY (Chinese Yuan), which is a moderate level among the general population in China [55]

^e^All participants, including those who had a zero value for the SSB category (consumers and non-consumers) and data were presented as mean (fifth percentile, ninety-fifth percentile) to obtain more information about various changes [33]

^f^Only participants with the category of SSB consumption (consumers) and data were presented as median (Q1, Q3)

^*^Significant differences between the categories of characteristics, tested using Student’s *t*-test for normally distributed continuous variables, Kruskal–Wallis *H* test for non-normally distributed continuous variables and chi-square test for categorical variables

### Association for SSB consumption between parents and children

The multivariable logistic analysis for the prospective associations for SSB intake between parents and offspring is presented in Table 2. In boys and girls, the chance of being an SSB consumer was significantly higher if their parents were SSB consumers.

**Table 2:** Odds ratios and 95% confidence intervals for children’s SSB intake by parental SSB consumption^1^

	Paternal SSB consumption		Maternal SSB consumption		Parental SSB consumption		
	Not consume	Consume	Not consume	Consume	Neither consumes	Only one consumes	Both consume
Boys’ SSB intake (*n* = 482)							
Basic model	1.0	5.3 (1.4, 14.2)^**^	1.0	2.4 (1.3, 4.5)^**^	1.0	2.4 (1.2, 5.0)^*^	5.0 (2.2, 13.4) ^**^
Adjusted model^b^	1.0	5.4 (2.4, 14.7)^**^	1.0	2.5 (1.3, 4.9)^**^	1.0	2.5 (1.2, 5.6) ^**^	5.3 (2.2, 14.7)^**^
Girls’ SSB intake (*n* = 422)							
Basic model	1.0	3.2 (1.6, 6.8)^**^	1.0	2.1 (1.2, 3.9)^*^	1.0	1.3 (0.7, 2.4)	4.5 (1.9, 12.1)^**^
Adjusted model^b^	1.0	3.2 (1.5, 7.2)^*^	1.0	2.0 (1.1, 4.0)^*^	1.0	1.2 (0.6, 2.4)	4.7 (1.9, 13.6)^*^

*SSB* sugar-sweetened beverage

^a^Values are odds ratios and 95% confidence intervals

^b^Adjusted for age of offspring, BMI *z*-score, household income, residency, sibship, paternal or maternal age, and paternal or maternal educational level

^*^P < 0.05, ^**^P < 0.005

For the difference between boys and girls, we found that odd ratios (ORs) for the association between boys’ SSB consumption and paternal/maternal SSB intake were 0.3–0.7 times higher than the ORs for the association between girls’ SSB consumption and parental/maternal SSB intake (paternal consumption: OR _for boys_ = 5.4 vs. OR _for girls_ = 3.2; maternal intake: OR _for boys_ = 2.5 vs. OR _for girls_ = 2.0). And for the difference between fathers and mothers, we found that the OR of fathers was 1.2 times higher than that of mothers among boys (OR _for fathers_ = 5.4 vs. OR _for mothers_ = 2.5) and 0.6 times higher among girls (OR _for fathers_ = 3.2 vs. OR _for mothers_ = 2.0).

Furthermore, compared with boys whose neither fathers nor mothers consumed SSB, boys with one parent drinking SSBs had a 2.5 times higher chance of consuming SSB, and a 5.3 times higher chance if two parents were SSB consumers. Compared with girls whose neither fathers nor mothers consumed SSB, the chance of consuming SSB was not different among girls with one parent drinking SSB, and the chance was 4.7 times higher among girls with two parents drinking SSB.

Sensitivity analyses showed no substantial changes in the results after excluding participants with missing values for the socio-demographic characteristics (Table S2). Similar results were also found in the association for amount of SSB consumption between parents and children (Table S3).

### Association for SSB consumption between siblings and children

Moreover, when parental SSB consumption was in the same condition, children who have sibling(s) were less likely to consume SSB than those without siblings (Figure S1; Table S4). There was an interaction between sibship and the associations of parental SSB intake with children’s SSB consumption (*P*_for interaction_ = 0.05). The impact of siblings’ SSB intake on children’s SSB consumption is presented in Table 3. The adjusted logistic regression model showed that the chance of being an SSB consumer increased if the elder sibling was an SSB consumer (OR = 4.9, 95% CI, 1.1–21.1). However, younger siblings’ SSB intake was not statistically relevant to children’s SSB consumption (*P*_for trend_ > 0.06).

**Table 3:** Odds ratios and 95% confidence intervals for children’s SSB intake by sibling’s SSB consumption^a^

	Sibling’s SSB consumption			
	Older sibling^b^		Younger sibling^c^	
	Not consume	Consume	Not consume	Consume
Basic model	1.0	5.6 (1.7, 18.4)^**^	1.0	3.0 (1.2, 7.5)^*^
Adjusted model^d^	1.0	4.9 (1.1, 21.1)^*^	1.0	2.8 (1.0, 8.1)

*SSB* sugar-sweetened beverage

^a^Values are odds ratios (OR) and 95% confidence intervals

^b^Sibling/child dyads: *n* = 141

^c^Sibling/child dyads: *n* = 124

^d^Adjusted for child sex, age difference, and parental SSB consumption

^*^P < 0.05, ^**^P < 0.005

## Discussion

In the present study, higher parental SSB consumption was prospectively associated with higher children’s SSB intake, and this association was more pronounced in boys than in girls, and fathers had a stronger impact than mothers. Additionally, children’s SSB consumption was influenced prospectively by their older siblings’ SSB intake rather than younger siblings’.

In the present study, children with parents who drink SSB were more likely to consume SSB, which was in line with the studies in 2–17 years old children and adolescents from the US [6, 9, 12], and 8–12 years old children from New Zealand [8]. Parents serve as role models for children’s behavior [5], and parental intake of vegetables and fruit, red meat, dairy and dairy products can directly affect children’s consumption of these foods and beverages [35, 36]. In addition, foods are mainly prepared and purchased by parents at home [37], and those with a diet rich in SSB might often store SSBs at home and may be less apt to restrict or have rules on their children’s SSB consumption. The availability and consumption of SSB for these children would, thus, be higher than those with parents who seldom drink SSB. Other than parental dietary behavior, previous researchers examined parental educational levels were examined as a factor in children’s SSB consumption [38]. Higher-educated parents tended to be well-off economically [39]. In this study, 88.2% of parents were high school graduates or below and two-thirds of households have a monthly personal income of less than 3200 Yuan (488.7 $). When parents consume SSB, the odds of children consuming SSB were lower in this study (OR:5.3; 95%CI: 1.4, 14.2) than in the US study (OR:8.9; 95%CI:4.6, 17.3) with a higher parental education level and a good socioeconomic status [12]. This contradiction might be explained by different SSB market prices. In developing countries, SSBs were usually less affordable than in western countries [40], and Chinese parents of better economic status may be the ones who regularly buy SSBs for their children, while in the United States, low-income parents often choose to feed their children with low-priced SSB [8]. Additionally, this study included a relatively large number of rural children. Supermarkets are far from their places of residence, and these families may go to the supermarket once in a very long time to make purchases. Some rural areas in China are located in mountainous areas with inconvenient transportation[41], and parents living in these areas would buy more household necessities rather than expensive and heavy SSBs. These would decrease the accessibility of SSB to children. Different amounts of SSB intake may also have contributed to the difference. In our study, the median SSB consumption of Chinese children was 70.3 ml/day, which was much lower than the mean SSB consumption of children in the USA (419.2 ml/day) [32].

Potential sex differences were found in intergenerational relationships between parents and their offspring [15, 16]. In this analysis, we paid special attention to the sex differences and found that boys’ SSB consumption was more susceptible to their parents' SSB intake than girls’, and fathers seemed to have a greater influence on whether children consume SSB than mothers. These differences may be explained by different attitudes towards consuming SSB between males and females, and diverse parenting styles in sons and daughters. First, different attitudes have been shown to produce different patterns of behavior [42]. In adults, the prevalence of sweetened soft drink consumption for men was higher by at least 5% than for women [17]. In children, boys tend to link the behavior of consuming SSB, especially carbonated beverages, with “popular”, “cool”, and “risky”, and have a positive attitude toward SSB [43–45]. When they witness others drinking SSB, to make themselves look cool and popular, they are more inclined to choose SSB rather than plain water. Girls, however, tend to link the behavior of consuming healthy beverages with good body shape and are more inclined to choose plain water and 100% juice instead of SSB [44, 46, 47]. Secondly, different parenting styles would lead to different parent–child intimacy communication styles [48]. In China, authoritative parenting styles were often found in boys’ families [49]. Boys in these families are more likely to imitate their parents’ behavior to reduce the possibility of being scolded for making mistakes. When witnessing parents drinking SSB, to imitate their behavior, boys may choose SSB. Democratic styles were often found in girls’ families [49], they are less likely to drink SSB to imitate parental behavior.

In this study, fathers seemed to have a greater influence on whether children consume SSB than mothers, which was inconsistent with a recent cross-sectional study in Chinese population [1]. The studies were of the same population, and socio-demographic parameters were relatively similar. The discrepancy between studies may be related to the different methodologies and different selections of participants. Selection of study participants and detained processing of children with multiple surveys were unknown in that study, the same child could be included as a study participant more than once. In Chinese population, fathers instead of mothers were proved to have an impact on children’s dietary protein intake [50], implying that a similar role may exist in SSB consumption. This hypothesis requires further investigation in longitudinal studies. While we do find that boys were 1.3–1.7 times more likely to be influenced by their parental SSB consumption than girls, the bias due to inevitable limitations challenges the validity of these findings, including observational nature of the study, selection bias, missing data and measurement error. However, this is the first study to focus on sex differences in SSB consumption in intergenerational relationships, these findings still provide an interesting perspective for future research in other populations, including a larger sample across Chinese general population.

In our study, children's SSB intake would be affected by older siblings’ SSB consumption rather than that of younger brothers or sisters, which was consistent with a recent cross-sectional study in England [13]. O’Leary et al. [13] only found a correlation of intrahousehold SSB consumption between children of different ages, and because of the methodological limitations, the direction of causality is difficult to assess. The results of this study further determined the direction of the association. In the present study, the older children were already in adolescence (median age: 12 years), and younger children were still in prepubertal period (median age: 8 years). Adolescents have considerable autonomy and decision-making power regarding their dietary behavior [51]. Compared to their younger siblings, they prefer spending lots of time with their friends [52] and get pocket money [8] which may be used to purchase unhealthy food. For younger children, they tend to imitate older siblings to develop their dietary behavior [53]. In 2016, China officially ended the one-child policy, and in 2021, the government even encouraged reproductive-age women to have three births. More than 8 million second child were born in 2016, and 59.5% of newborns were second child in 2019 [54]. With the increasing number of multiple-child families in China, the influence of sibling’s effect cannot be ignored. For second child’s parents, who were likely raised in a single-child family and have little experience with siblings, and this makes education for multiple children has become a critical social issue requiring immediate attention. However, due to limitations imposed by the small sample size, our estimation results for sex differences in the relationships between siblings and children in SSB consumption were not reliably available (Table S5), still, our findings provided an interesting new direction for future research in dietary consumption.

Our study has several strengths, including its prospective nature to establish causal order, and the ability to adjust for a number of potential confounders both in children and in parents. A further advantage lies in the detailed exploration of SSB consumption in both fathers and mothers. Moreover, unlike other studies of children or adolescents, we noted sex differences in SSB intergenerational relationships, which may require more attention in the development of SSB reduction policies for children.

Nevertheless, some limitations should also be mentioned. First, though the amount of SSB intake in this study is consistent with that obtained in other studies of Chinese children and adults, the dietary assessment method of SSB has not been validated yet. And self-report of dietary data and the unavailability of energy intake data obtained through the food frequency questionnaire introduced the possibility of bias and underreporting. Second, as the CHNS has not regularly tested the consistency in the operation of investigators, there might be inadequacies with the quality of basic anthropometric measurements (height and weight). Third, though acceptance of initial invitation for study participants varied by area, we excluded participants who refused participation, which may have resulted in the selection of a more “health-conscious” study sample. Fourth, the sample size limited the extent of analysis which makes us unable to analyze the sex difference in siblings’ impact and in families with only one parent drinking SSB.

In conclusion, our study illustrated that parental SSB consumption and older siblings’ SSB intake, not younger siblings’ SSB intake, were prospectively associated with Chinese children’s SSB consumption. And in Chinese families, boys were more susceptible to their parents' SSB intake than girls, and fathers seemed to have more influence on children’s SSB consumption than mothers.

## Supplementary Information

Below is the link to the electronic supplementary material.

Supplementary file1 (DOCX 58 KB)

## Data Availability

The China Health and Nutrition Survey is an open-access resource, data are available at http://www.cpc.unc.edu/projects/china/data/datasets.
