# Supplementary material for: Prospective association of family members’ sugar-sweetened beverages intake with children’s sugar-sweetened beverages consumption in China
Source: Eur J Nutr. 2022 Aug 5;62(1):175–84. doi: 10.1007/s00394-022-02971-3 (PMC9899727; doi:10.1007/s00394-022-02971-3)
Supplement: Supplementary file 1 — Supplementary file1 (DOCX 58 KB) [file 394_2022_2971_MOESM1_ESM.docx]

**Supplemental Table 1** Descriptive Statistics of Sociodemographic characteristics and SSB consumption of Children Included in (vs Excluded From) the Primary Analytic Sample^1^

| Characteristics | Included sample | Excluded sample | *P* ^*^ |
| --- | --- | --- | --- |
| n (%) | 904 | 1324 | - |
| **Socio-demographic issues** |  |  |  |
| Age at baseline (years) | 12 (9, 14) | 12 (8, 14) | 0.7 |
| BMI z-score^2^ | -0.4 (1.2) | -0.34 (1.1) | 0.08 |
| Single-child^3^ [n (%)] | 655 (72.5) | 1125 (84.9) | <0.01 |
| Paternal age (years) | 39 (36, 42) | 39 (36, 43) | 0.1 |
| Maternal age (years) | 38 (35, 41) | 39 (36, 43) | 0.02 |
| High monthly personal income^4^ [n (%)] | 307 (34.0) | 456 (34.5) | 0.46 |
| Live in urban area [n (%)] | 305 (33.7) | 484 (36.6) | 0.2 |
| **Children’s SSB consumption** [n (%)] | 792 (87.6) | 1149 (86.8) | 0.6 |
| **Paternal SSB consumption** [n (%)] | 331 (36.6) | 442 (33.4) | 0.1 |
| **Maternal SSB consumption** [n (%)] | 437 (48.3) | 597 (45.1) | 0.1 |

*SSB* sugar-sweetened beverage

^1^ Values are means (SD) or medians (Q1, Q3) or frequencies

^2^ BMI z-score, body mass index z-score calculated according to the Chinese reference curves[28]

^3^ Single-child, children from families with only one child

^4^ Monthly personal income at least ≥3200 CNY (Chinese Yuan), which is a moderate level among the general population in China[59]

*Significant differences between the categories of characteristics, tested using student t-test for normally distributed continuous variables, Kruskal-Wallis H test for non-normally distributed continuous variables and chi-square test for categorical variables

**Supplemental Table 2** Odds ratios and 95% confidence intervals for children’s SSB intake by parental SSB consumption in participants without missing values on covariates ^1^

|  | Paternal SSB consumption | |  | Maternal SSB consumption | |  | Parental SSB consumption | | |
| --- | --- | --- | --- | --- | --- | --- | --- | --- | --- |
|  | Not consume | Consume |  | Not consume | Consume |  | Neither consumes | Only one consumes | Both  consume |
| **Boys’ SSB intake** (n=482) |  |  |  |  |  |  |  |  |  |
| Basic model | 1.0 | 4.9 (2.2, 12.9) ^**^ |  | 1.0 | 2.4 (1.3, 4.5) ^**^ |  | 1.0 | 2.5 (1.2, 5.4) ^*^ | 4.6 (2.0, 12.4) ^**^ |
| Adjusted model^2^ | 1.0 | 4.8 (2.1, 13.0) ^**^ |  | 1.0 | 2.3 (1.2, 4.6) ^*^ |  | 1.0 | 2.5 (1.2, 5.8) ^*^ | 4.6 (1.9. 12.9) ^**^ |
| **Girls’ SSB intake** (n=422) |  |  |  |  |  |  |  |  |  |
| Basic model | 1.0 | 2.9 (1.4, 6.5) ^**^ |  | 1.0 | 2.0 (1.1, 3.9) ^*^ |  | 1.0 | 1.3 (0.7, 2.8) | 3.8 (1.6, 10.3) ^**^ |
| Adjusted model^2^ | 1.0 | 3.2 (1.5, 7.6) ^*^ |  | 1.0 | 2.2 (1.1, 4.5) ^*^ |  | 1.0 | 1.3 (0.6, 3.0) | 4.5 (1.8, 13.1) ^**^ |

*SSB* sugar-sweetened beverage

^1^ Values are odds ratios and 95% confidence intervals

^2^ Adjusted for age of offspring, BMI z-score, household income, residency, sibship, paternal or maternal age, and paternal or maternal educational level

^*^ *P*＜0.05, ^**^*P*＜0.005

**Supplemental Table 3** Associations between amounts of parental SSB consumption and amounts of children’s SSB intake across sex in SSBs consumers^1^

|  | Paternal SSB consumption | | |  | Maternal SSB consumption | | |
| --- | --- | --- | --- | --- | --- | --- | --- |
|  | *β* | SE | *P* _trend_ |  | *β* | SE | *P* _trend_ |
| **Boys’ SSB intake**^2^ |  |  |  |  |  |  |  |
| Basic model | 1.73 | 0.38 | <0.01 |  | 2.03 | 0.33 | <0.01 |
| Adjusted model^4^ | 1.86 | 0.41 | <0.01 |  | 1.98 | 0.33 | <0.01 |
| **Girls’** **SSB intake**^3^ |  |  |  |  |  |  |  |
| Basic model | 0.40 | 0.18 | 0.02 |  | 1.11 | 0.39 | 0.01 |
| Adjusted model^4^ | 0.35 | 0.19 | 0.06 |  | 0.64 | 0.40 | 0.1 |

*SSB* sugar-sweetened beverage, *SE* standard error

^1^ Values are presented as regression coefficients (*β*) and SE obtained by linear regression analysis

^2^ Father/boy dyads: n=108; Mother/boy dyads: n=135

^3^ Father/girl dyads: n=97; Mother/girl dyads: n=106

^4^ Adjusted for age of offspring, BMI z-score, household income, residency, sibship, paternal or maternal age, and paternal or maternal educational level

Both consume

Only one consumes

Neither consumes

Parental SSB consumption

Percentage of SSB

consumers of offspring (%)

0.0

*P* _for interaction_: 0.05

Girl

Girl

Boy

Boy

Child with sibling(s)

Only child

**Supplemental Fig.1** Association between children’s SSB intake and parental SSB consumption by the categories of sibship and sex of offspring (n=904). Data are percentage of SSB consumers of offspring. *P* for interactions refers to the *p* values obtained for the interaction between parental SSB consumption and sibship in the association with children’s SSB intake. Participants in groups: both parents consume, only child, boy: n=96; both parents consume, only child, girl: n=86; both parents consume, child with sibling(s), boy: n=46; both parents consume, child with sibling(s), girl: n=39; one parent consumes, only child, boy: n=92; one parent consumes, only child, girl: n=81; one parent consumes, child with sibling(s), boy: n=37; one parent consumes, child with sibling(s), girl: n=24; neither of parents consumes, only child, boy: n=157; neither of parents consumes, only child, girl: n=143; neither of parents consumes, child with sibling(s), boy: n=54; neither of parents consumes, child with sibling(s), girl: n=48. *See* Table S2 for information on *P* value in different sub-groups

**Supplemental Table 4** Differences in the percentage of SSB consumers between single child and those with sibling(s) stratified by sex and parental SSB consumption^1^

|  | single child | Child with sibling(s) | *P^*^* |
| --- | --- | --- | --- |
| Both parents consume |  |  |  |
| Boy | 92 (95.8) | 44 (95.7) | 0.6 |
| Girl | 81 (94.2) | 38 (97.4) | 0.4 |
| Only one parent consumes |  |  |  |
| Boy | 88 (95.7) | 30 (81.1) | 0.01 |
| Girl | 70 (86.4) | 19 (79.2) | 0.5 |
| Neither of parent consumes |  |  |  |
| Boy | 131 (83.4) | 42 (77.8) | 0.2 |
| Girl | 122 (85.3) | 34 (70.8) | 0.02 |

^1^ Values are frequency [n (%)]

* The differences between the categories were tested using Fisher’s exact test

**Supplemental Table 5** Odds ratios and 95% confidence intervals for children’s SSB intake by sibling’s SSB consumption^1^

|  | Older sibling’s SSB consumption | | | | |  | younger sibling’s SSB consumption | | | | |
| --- | --- | --- | --- | --- | --- | --- | --- | --- | --- | --- | --- |
|  | Brother’s SSB consumption | |  | Sister’s SSB consumption | |  | Brother’s SSB consumption | |  | Sister’s SSB consumption | |
|  | Not consume | Consume |  | Not consume | Consume |  | Not consume | Consume |  | Not consume | Consume |
| **Boys’ SSB intake** |  |  |  |  |  |  |  |  |  |  |  |
| Basic model | 1.0 | 4.0 (0.5, 29.2) |  | 1.0 | 1.2 (0.1, 10.3) |  | 1.0 | 3.4 (0.5, 22.6) |  | 1.0 | 0.8 (0.1, 8.1) |
| Adjusted model ^2^ | 1.0 | 6.2 (0.5, 85.1) |  | 1.0 | 2.9 (0.1, 86.8) |  | 1.0 | 3.3 (0.4, 25.5) |  | 1.0 | 1.4 (0.1, 19.4) |
| **Girls’ SSB intake** |  |  |  |  |  |  |  |  |  |  |  |
| Basic model | 1.0 | <0.001 |  | 1.0 | >999.999 |  | 1.0 | 1.9 (0.4, 7.9) |  | 1.0 | >999.999 |
| Adjusted model ^2^ | 1.0 | <0.001 |  | 1.0 | >999.999 |  | 1.0 | 1.7 (0.3, 9.5) |  | 1.0 | >999.999 |

*SSB* sugar-sweetened beverage

^1^ Values are odds ratios (OR) and 95% confidence intervals

^2^ Adjusted for child sex, age difference, and parental SSB consumption

^*^ *P*＜0.05, ^**^*P*＜0.005

**Supplemental Table 6** Differences in baseline characteristics between children who consume SSB and children who do not consume^1^

| Characteristics | Consume SSB | Not consume SSB | *P* ^*^ |
| --- | --- | --- | --- |
| n (%) | 792 (87.6) | 112 (12.4) |  |
| **Socio-demographic issues** |  |  |  |
| Age at baseline (years) | 12 (9, 14) | 12 (9, 14) | 0.3 |
| BMI z-score^2^ | -0.5 (1.2) | -0.2 (0.9) | <0.01 |
| Single-child^3^ [n (%)] | 208 (26.3) | 41 (36.6) | 0.02 |
| Paternal age (years) | 39 (36, 42) | 39 (36, 42) | 0.9 |
| Maternal age (years) | 38 (35, 41) | 37 (35, 41) | 0.9 |
| High monthly personal income^4^ [n (%)] | 260 (33.4) | 27 (25.5) | 0.2 |
| Live in urban area [n (%)] | 276 (34.9) | 29 (25.9) | 0.06 |
| Parental education duration >12 years [n (%)] | 96 (12.1) | 11 (9.8) | 0.5 |

*SSB* sugar-sweetened beverage

^1^ Values are means (SD) or medians (Q1, Q3) or frequencies

^2^ BMI z-score, body mass index z-score calculated according to the Chinese reference curves[28]

^3^ Single-child, children from families with only one child

^4^ Monthly personal income at least ≥3200 CNY (Chinese Yuan), which is a moderate level among the general population in China[59]

*Significant differences between the categories of characteristics, tested using student t-test for normally distributed continuous variables, Kruskal-Wallis H test for non-normally distributed continuous variables and chi-square test for categorical variables
